
JOURNAL INFORMATION
==============================
NLM Title Abbreviation: Sci China Life Sci
Journal ID: Sci China Life Sci
NLM Journal ID: 101529880

Science China. Life Sciences

ISSN: 1674-7305
EISSN: 1869-1889

ARTICLE INFORMATION
==============================
PMCID: PMC7088662
PMID: 25239448
DOI: 10.1007/s11427-014-4756-5
Article ID: 4756
Article version: 1
Subjects: Insight

Potential clinical treatment for Ebola pandemic

Zhong Ying 1
Xu Jun 2
Li TaiSheng 3
Yu XueZhong 2
Sheng MiaoMiao shengmm@aliyun.com
4
1 grid.12527.33 0000000106623178 State Key Laboratory of Medical Molecular Biology, Institute of Basic Medical Sciences, Chinese Academy of Medical Sciences; Department of Biochemistry and Molecular Biology, Peking Union Medical College, Tsinghua University, Beijing, 100005 China
2 grid.410318.f 0000000406323409 Department of Emergency Medicine, Peking Union Medical Collage Hospital, Peking Union Medical College, Chinese Academy of Medical Sciences, Beijing, 100730 China
3 grid.410318.f 0000000406323409 Department of Infectious Diseases, Peking Union Medical College Hospital, Peking Union Medical College, Chinese Academy of Medical Sciences, Beijing, 100730 China
4 grid.218292.2 000000008571108X Laboratory of Molecular Genetics of Aging & Tumor, Medical Faculty, Kunming University of Science and Technology, Chenggong Campus, Kunming, 650500 China
Electronic publication date: 2014 Sep 19
Print publication date: 2014
Volume: 57
Issue: 10
First page: 982
Last page: 984
Received 2014 Sep 14; Accepted 2014 Sep 17
Copyright: © The Author(s) 2014
License: Open AccessThis article is distributed under the terms of the Creative Commons Attribution 4.0 International License (https://creativecommons.org/licenses/by/4.0), which permits use, duplication, adaptation, distribution, and reproduction in any medium or format, as long as you give appropriate credit to the original author(s) and the source, provide a link to the Creative Commons license, and indicate if changes were made.
License URL: https://creativecommons.org/licenses/by/4.0/

Keywords: Dronedarone, West African Country, Phosphorodiamidate Morpholino, Epidemic Hemorrhagic Fever, Vascular Barrier Integrity
issue-copyright-statement: © Science in China Press and Springer-Verlag GmbH 2014

==============================

[1]*State Key Laboratory of Medical Molecular Biology, Institute of Basic Medical Sciences, Chinese Academy of Medical Sciences; Department of Biochemistry and Molecular Biology, Peking Union Medical College, Tsinghua University; Beijing 100005, China;*
[2]*Department of Emergency Medicine, Peking Union Medical Collage Hospital, Peking Union Medical College, Chinese Academy of Medical Sciences, Beijing 100730, China;*
[3]*Department of Infectious Diseases, Peking Union Medical College Hospital, Peking Union Medical College, Chinese Academy of Medical Sciences, Beijing 100730, China;*
[4]*Laboratory of Molecular Genetics of Aging & Tumor, Medical Faculty, Kunming University of Science and Technology, Chenggong Campus, Kunming 650500, China*

West African countries are currently suffering the most severe pandemic of Ebola virus disease ever recorded, with 4269 human cases and 2288 deaths (through 6 September 2014), and the number of cases in the current outbreak exceeds the number from all previous outbreaks combined. Ebola virus disease was first described in 1976 originating from the Ebola River in the Democratic Republic of the Congo. Five Ebola viruses have been reported to cause Ebola virus pandemics, including Zaire Ebola virus, Sudan Ebola virus, Ivory Coast Ebola virus, Reston Ebola virus and Bundibugyo Ebola virus. The Zaire Ebola virus is the most deadly with a fatality rate up to 90%. Unfortunately, the current outbreak in the West African countries is caused by the Zaire Ebola virus, and no specific treatment for the disease is yet available.

Like other filoviruses, Ebola virus has the special thread-like structure seen on electronic micrographs. Ebola virion contains a linear, single-stranded negative-sense RNA genome, which is approximately 19,000 nucleotides in length without a polyadenylated 3′ terminus or a 5′ capped end. The genome encodes seven structural proteins and one non-structural protein ordered 3′–leader–nucleoprotein (NP)–viral protein VP35–VP40–glycoprotein (GP)/secreted GP (sGP)–VP30–VP24–RNA-dependent RNA polymerase (L)–trailer–5′. The leader and trailer are non-transcribed regions but are important signals that control the transcription, replication and packaging of the viral genomes into new virions. Glycoprotein (GP) is the main viral determinant of Ebola virus pathogenicity.

The outbreaks of Ebola virus disease have been primarily restricted to Africa. This restriction may be because Ebola virus disease kills infectors at a high mortality rate and governments and individuals always respond quickly to quarantine these areas. The natural reservoir(s) and primary transmission route(s) of Ebola virus disease are not entirely clear. It is reported that bats could be a possible reservoir, and they may support the replication of Ebola virus without symptoms of the disease. Ebola virus disease is believed to occur after an Ebola virus is transmitted to a human via contact with an infected animal. Human-to-human transmission appears to occur through direct contact with blood or other body fluids from infected patients or by contact with contaminated medical equipment, so barrier nursing is necessary to limit the spread of infection. A report from Natural News confirmed that Ebola can be transmitted through aerosols and that the virus can survive for days outside a host (http://www.naturalnews.com).

The incubation period of Ebola virus ranges from 2 to 21 days (http://www.cdc.gov/vhf/ebola/symptoms/index.html).

The abrupt onset of Ebola hemorrhagic fever resembles the common flu with clinical symptoms, such as fever, headache, sore throat, weakness, myalgia, abdominal pain, diarrhea, and vomiting. Haemorrhagic manifestations arise during the peak of the illness. The liver, kidney, and spleen are damaged by the Ebola virus, leading to serious bleeding and coagulation abnormalities, including gastrointestinal bleeding and a range of hematological irregularities, such as lymphopenia and neutrophilia. When the virus attacks reticuloendothelial cells, cytokines are over produced and cause an exaggerated inflammatory response that is not protective. The microvascular endothelial cells are eventually infected by the Ebola virus, and the vascular integrity is destroyed. The terminal stages of Ebola virus infection usually include diffuse bleeding, and hypotensive shock accounts for many Ebola virus fatalities.

Until now, we have had no effective vaccines or treatments for Ebola virus. Therefore, comprehensive treatment including supportive therapy is most important, such as balancing fluids and electrolytes, maintaining oxygen levels, and injecting anticoagulants. However, several prospective treatment methods are being studied. rNAPc2, nematode-derived anticoagulation protein, has shown 33% efficacy in the treatment of macaque infected with Zaire Ebola virus. It plays an important role in weakening coagulation and inflammatory [1]. T-750 (favipiravir) is a pyrazinecarboxamide derivative and inhibits influenza virus replication. T-750 is being tested in the late clinical stage, and it seems to effectively treat Ebola virus disease in a mouse model. Favipiravir can be converted to T-705-ribofuranosyl-50-triphosphate, a nucleotide analog that can inhibit the viral RNA-dependent RNA polymerase or induce lethal mutagenesis by incorporating into the viral RNA [2]. BCX-4430, a new nucleotide analogues, inhibits virus replication by inhibiting viral RNA polymerase function. It exhibits broad-spectrum antiviral activity against numerous viruses, including flaviviruses, bunyaviruses, and coronaviruses [3]. The serum of recovered patients may also be useful to develop a treatment for Ebola virus disease. During this outbreak of Ebola virus in the West African countries, two Americans became infected, and they benefited from a cocktail antibody treatment, called ZMapp, which is a mixture of three humanized mouse monoclonal antibodies (c13C6, h-13F6, and c6D8). The three anti-Ebola virus monoclonal antibodies had significantly protected rhesus macaques from a lethal challenge of Ebola virus [4]; however, no human safety studies were performed before the drug was administered to these two patients. The FDA has approved ZMapp for the treatment of patients infected with Ebola virus in the current emergency situation. The antisense technology can also be used to treat Ebola virus disease. Warren et al. [5] reported that small interfering RNAs and phosphorodiamidate morpholino oligomers may effectively prevent Ebola virus disease in nonhuman primates by targeting the Zaire Ebola virus RNA polymerase L protein. Based on this technology, TKM-Ebola has also been approved by the FDA to treat patients infected with Ebola virus. However, these experimental treatments have no recorded human safety trials. Some drugs approved for human use for other indications, including chloroquine [6], estrogen receptor (clomiphene and toremifene) [7], ion channel blocker (amiodarone, dronedarone and verapamil) [8] and imatinib [6], have shown activity against Ebola virus infection or entry *in vitro* or in rodent models. These drugs would be candidates for treating EHF, alone or in combination with other antiviral drugs.

The common, broad-spectrum, antiviral drug Ribavirin might only be effective among moderately ill patients, similar to Crimean-Congo hemorrhagic fever (CCHF). Ribavirin is a synthesized guanosine analog that may inhibit RNA-dependent RNA polymerase, mutagenize the genome and inhibit inosine monophosphate dehydrogenase (IMPDH), which is a key enzyme for *de novo* purine nucleotide biosynthesis [9]. In addition to anti-viral treatments, we could also consider medicines that provide vascular protection. Rutin, or vitamin P, is a major flavonoid that can be used in CCHF and epidemic hemorrhagic fever to protect vascular barrier integrity. Cell adhesion molecules (CAMs), including selectin, intercellular adhesion molecule-1 (ICAM-1) and vascular cell adhesion molecule-1 (VCAM-1), play critical roles in cell adhesion to the vascular endothelium resulting in extravasation of cells and vascular inflammation. Rutin can inhibit the expression of CAMs, thus inhibiting the adhesion and migration of leukocytes and affecting vascular barrier integrity [10,11]. Vitamin C, or ascorbic acid, can also be used in CCHF and epidemic hemorrhagic fever [11]. It may prevent the endothelial dysfunction via stimulating endothelial proliferation, modulating blood flow by sparing endothelial cell-derived nitric oxide and increasing the level of type IV collagen in the basement membrane [12]. High-dose methylprednisolone also effectively treats patients with CCHF [13]. Activation of NF-κB could upregulate the proinflammatory cytokines followed by endothelial cell dysfunction. Research showed that blockade of NF-κB activation preserved endothelial barrier integrity. Patel et al. [14] reported that statins had the role of anti-inflammatory and immunomodulatory by downregulating expression levels of NF-κB, sCD40L and sICAM. In addition, it can also decrease expression of tissue factor and thrombin and increase activation of thrombomodulin [15]. Currently, statins have been used in the treatment of cardiovascular disease, which could be considered for treating patients with EHF.

Ebola virus disease is a painful reminder, all individuals who have been to the infected area should be isolated for at least two weeks. To prepare for the Ebola virus pandemic, besides detection materials, we should store enough drugs specific to Ebola virus disease and commonly used drugs for hemorrhagic fever.

**Open Access** This article is distributed under the terms of the Creative Commons Attribution License which permits any use, distribution, and reproduction in any medium, provided the original author(s) and source are credited.

This article is published with open access at link.springer.com


References

1 Geisbert TW Hensley LE Jahrling PB Larsen T Geisbert JB Paragas J Young HA Fredeking TM Rote WE Vlasuk GP Treatment of Ebola virus infection with a recombinant inhibitor of factor VIIa/tissue factor: a study in rhesus monkeys Lancet 2003 362 1953 1958 10.1016/S0140-6736(03)15012-X 14683653
2 Oestereich L Ludtke A Wurr S Rieger T Munoz-Fontela C Gunther S Successful treatment of advanced Ebola virus infection with T-705 (favipiravir) in a small animal model Antiviral Res 2014 105 17 21 10.1016/j.antiviral.2014.02.014 24583123
3 Warren TK Wells J Panchal RG Stuthman KS Garza NL Van Tongeren SA Dong L Retterer CJ Eaton BP Pegoraro G Honnold S Bantia S Kotian P Chen X Taubenheim BR Welch LS Minning DM Babu YS Sheridan WP Bavari S Protection against filovirus diseases by a novel broad-spectrum nucleoside analogue BCX4430 Nature 2014 508 402 405 10.1038/nature13027 24590073 PMC7095208
4 Olinger GG Jr. Pettitt J Kim D Working C Bohorov O Bratcher B Hiatt E Hume SD Johnson AK Morton J Pauly M Whaley KJ Lear CM Biggins JE Scully C Hensley L Zeitlin L Delayed treatment of Ebola virus infection with plant-derived monoclonal antibodies provides protection in rhesus macaques Proc Natl Acad Sci USA 2012 109 18030 18035 10.1073/pnas.1213709109 23071322 PMC3497800
5 Warren TK Warfield KL Wells J Swenson DL Donner KS Van Tongeren SA Garza NL Dong L Mourich DV Crumley S Nichols DK Iversen PL Bavari S Advanced antisense therapies for postexposure protection against lethal filovirus infections Nat Med 2010 16 991 994 10.1038/nm.2202 20729866
6 Uebelhoer LS Albarino CG McMullan LK Chakrabarti AK Vincent JP Nichol ST Towner JS High-throughput, luciferase-based reverse genetics systems for identifying inhibitors of Marburg and Ebola viruses Antiviral Res 2014 106 86 94 10.1016/j.antiviral.2014.03.018 24713118
7 Johansen LM Brannan JM Delos SE Shoemaker CJ Stossel A Lear C Hoffstrom BG Dewald LE Schornberg KL Scully C Lehar J Hensley LE White JM Olinger GG FDA-approved selective estrogen receptor modulators inhibit Ebola virus infection Sci Translat Med 2013 5 190ra179 10.1126/scitranslmed.3005471 PMC3955358 23785035
8 Gehring G Rohrmann K Atenchong N Mittler E Becker S Dahlmann F Pohlmann S Vondran FW David S Manns MP Ciesek S von Hahn T The clinically approved drugs amiodarone, dronedarone and verapamil inhibit filovirus cell entry J Antimicrob Chemother 2014 69 2123 2131 10.1093/jac/dku091 24710028 PMC7110251
9 Dokuzoguz B Celikbas AK Gok SE Baykam N Eroglu MN Ergonul O Severity scoring index for crimean-congo hemorrhagic fever and the impact of ribavirin and corticosteroids on fatality Clin Infect Dis 2013 57 1270 1274 10.1093/cid/cit527 23946218
10 Lee W Ku SK Bae JS Barrier protective effects of rutin in LPS-induced inflammation in vitro and in vivo Food Chem Toxicol 2012 50 3048 3055 10.1016/j.fct.2012.06.013 22721984
11 Whitehouse CA Crimean-Congo hemorrhagic fever Antiviral Res 2004 64 145 160 10.1016/S0166-3542(04)00163-9 15550268
12 May JM Harrison FE Role of vitamin C in the function of the vascular endothelium Antioxid Redox Signal 2013 19 2068 2083 10.1089/ars.2013.5205 23581713 PMC3869438
13 Sharifi-Mood B Alavi-Naini R Metanat M Mohammadi M Shakeri A Amjadi A Efficacy of high-dose methylprednisolone in patients with Crimean-Congo haemorrhagic fever and severe thrombocytopenia Trop Doct 2013 43 49 53 10.1177/0049475513486642 23796671
14 Patel JM Snaith C Thickett DR Linhartova L Melody T Hawkey P Barnett AH Jones A Hong T Cooke MW Perkins GD Gao F Randomized double-blind placebo-controlled trial of 40 mg/day of atorvastatin in reducing the severity of sepsis in ward patients (asepsis trial) Crit Care 2012 16 R231 10.1186/cc11895 23232151 PMC3672620
15 Undas A Brummel-Ziedins KE Mann KG Anticoagulant effects of statins and their clinical implications Thromb Haemost 2014 111 392 400 10.1160/TH13-08-0720 24285296
